# Belimumab efficacy in mucocutaneous manifestations of systemic lupus erythematosus: a large *post hoc* analysis of five phase III clinical trials

**DOI:** 10.1093/rheumatology/keaf145

**Published:** 2025-03-14

**Authors:** Giorgia Grosso, Nefeli Giannopoulou, Alexander Tsoi, Nursen Cetrez, Dionysis Nikolopoulos, Julius Lindblom, Ioannis Parodis

**Affiliations:** Division of Rheumatology, Department of Medicine Solna, Karolinska Institutet, Karolinska University Hospital, and Center for Molecular Medicine (CMM), Stockholm, Sweden; Division of Rheumatology, Department of Medicine Solna, Karolinska Institutet, Karolinska University Hospital, and Center for Molecular Medicine (CMM), Stockholm, Sweden; Division of Rheumatology, Department of Medicine Solna, Karolinska Institutet, Karolinska University Hospital, and Center for Molecular Medicine (CMM), Stockholm, Sweden; Division of Rheumatology, Department of Medicine Solna, Karolinska Institutet, Karolinska University Hospital, and Center for Molecular Medicine (CMM), Stockholm, Sweden; Division of Rheumatology, Department of Medicine Solna, Karolinska Institutet, Karolinska University Hospital, and Center for Molecular Medicine (CMM), Stockholm, Sweden; Division of Rheumatology, Department of Medicine Solna, Karolinska Institutet, Karolinska University Hospital, and Center for Molecular Medicine (CMM), Stockholm, Sweden; Division of Rheumatology, Department of Medicine Solna, Karolinska Institutet, Karolinska University Hospital, and Center for Molecular Medicine (CMM), Stockholm, Sweden; Faculty of Medicine and Health, Department of Rheumatology, Örebro University, Örebro, Sweden

**Keywords:** SLE, skin, therapeutics, biologics, belimumab

## Abstract

**Objective:**

To determine the efficacy of belimumab on mucocutaneous manifestations of SLE in a large integrative analysis.

**Methods:**

Using data from five phase III clinical trials (BLISS-52; BLISS-76; BLISS-NEA; EMBRACE; BLISS-SC; *N* = 3086), we investigated the effect of belimumab vs. placebo on top of standard therapy on inducing improvement in mucocutaneous BILAG (mcBILAG) and mucocutaneous SLE Disease Activity Index 2000 (mcSLEDAI-2K), and on preventing mcBILAG flares. We employed logistic and Cox regression analysis, adjusting for trial variance.

**Results:**

Belimumab was superior to placebo in inducing mcBILAG (week-52 OR: 1.29; 95% CI: 1.07–1.57; *P =* 0.008) and mcSLEDAI-2K (week-52 OR: 1.37; 95% CI: 1.16–1.62; *P<* 0.001) improvement, as well as in inducing sustained (≥2 visits, maintained through week 52) mcBILAG (HR: 1.23; 95% CI: 1.07–1.41; *P* = 0.003) and mcSLEDAI-2K (HR: 1.24; 95% CI: 1.17–1.31; *P* < 0.001) improvement. These associations held true for patients with SLEDAI-2K ≥10 and positive anti-dsDNA levels at baseline, but not their counter groups. Belimumab prevented mcBILAG flares to a greater extent than placebo in patients with positive anti-dsDNA levels (HR: 0.70; 95% CI: 0.50–0.98; *P* = 0.035) and with a near-significant separation in patients with baseline SLEDAI-2K ≥10 (HR: 0.71; 95% CI: 0.51–1.00; *P* = 0.050), whereas no difference was seen in their counter groups.

**Conclusion:**

Belimumab is superior to placebo in inducing improvement and in preventing flares in the mucocutaneous domain of SLE, especially in patients with high disease activity and in serologically active patients.

Rheumatology key messages

- Belimumab is superior to placebo in inducing mucocutaneous improvement, measured by BILAG and SLEDAI-2K
- Belimumab is superior to placebo in inducing sustained improvement in the mucocutaneous SLE domain
- Belimumab is superior to placebo in preventing mucocutaneous flares in serologically active SLE patients

## Introduction

SLE is a systemic autoimmune disease known for its heterogeneity in clinical manifestations and immunological aberrancies [1, 2]. B lymphocytes play a pivotal role in autoantibody production, release of cytokines, and antigen presentation, contributing to the pathogenesis of SLE [3]. In mucocutaneous SLE, histopathology and immunofluorescence have shown deposition of immune complexes and complement components along the dermoepidermal junction, leading to tissue injury, vascular, and perivascular inflammation, and chronic mononuclear cell infiltration [4].

Belimumab is a biologic agent approved for the treatment of SLE. It is a fully human IgG1λ monoclonal antibody that specifically targets the soluble form of B cell activating factor belonging to the tumour necrosis factor family, also known as B lymphocyte stimulator [5, 6]. The efficacy of belimumab in addition to standard therapy (ST) in patients with SLE has been evaluated in several randomized, placebo-controlled, multicentre, double-blind phase III trials, including BLISS-52, a 52-week trial of intravenous (iv) belimumab conducted in Eastern Europe, Asia-Pacific and South America [7], BLISS-76, a 76-week trial of iv belimumab conducted in North America and Europe [8], BLISS Northeast Asia (NEA), a 52-week trial of iv belimumab conducted in Northeast Asia [9], EMBRACE, a 52-week trial of iv belimumab in SLE patients of self-identified black race, conducted in Brazil, Colombia, France, South Africa, the UK, and the US [10], and BLISS-subcutaneous (SC), a 52-week trial of subcutaneous (sc) belimumab conducted in North, Central, and South America, Eastern and Western Europe, Australia and Asia [11]. Moreover, real-world observational studies have corroborated the efficacy of belimumab in multiple clinical settings [12–17].

Current treatment recommendations for SLE are mainly guided by the involved organ systems [18], although manifestation-specific literature is scarce. In this study, we performed a *post hoc* analysis of compiled data from the clinical trials mentioned above to specifically quantify the effect of belimumab on mucocutaneous manifestations of SLE in terms of improvement, sustained improvement, and prevention of flares and thereby inform recommendations for the management of SLE.

## Materials and methods

### Study design and population

We designed a *post hoc* analysis of data from 3086 patients with SLE who participated in the phase III clinical trials of belimumab BLISS-52 (NCT00424476; *N* = 865) [7], BLISS-76 (NCT00410384; *N* = 819) [8], BLISS-NEA (NCT01345253; *N* = 677) [9], EMBRACE (NCT0163224; *N* = 448) [10], and BLISS-SC (NCT01484496; *N* = 836) [11]. All patients were ≥18 years old and fulfilled the revised ACR criteria for classification of SLE [19, 20]. At screening, all patients had an ANA titer ≥1:80 and/or serum anti-dsDNA antibody level ≥30 IU/ml and a Safety of Estrogens in Lupus National Assessment (SELENA) SLE Disease Activity Index (SLEDAI) score ≥6 in BLISS-52 and BLISS-76 or ≥8 in BLISS-SC, BLISS-NEA and EMBRACE. Patients with severe active central nervous system involvement or severe active lupus nephritis were excluded. All patients were receiving stable background non-biological ST that could include glucocorticoids, antimalarial agents, and immunosuppressants for at least 30 days before commencement of the trial intervention.

Upon randomization, patients received placebo or belimumab iv 1 mg/kg or iv 10 mg/kg in BLISS-52 and BLISS-76 and iv 10 mg/kg in BLISS-NEA and EMBRACE at baseline, week 2, week 4, and, thereafter, every fourth week in BLISS-52, BLISS-76, BLISS-NEA, and EMBRACE, or sc belimumab 200 mg/week in BLISS-SC, on top of ST. In our analysis, we excluded patients treated with iv belimumab 1 mg/kg (*N* = 559) and only considered the first 52 weeks of follow-up from all trials.

### Ethics

Data from the trials were made available by GlaxoSmithKline (GSK; Uxbridge, UK) through the Clinical Study Data Request (CSDR) platform. Written informed consent was obtained from all study participants prior to enrolment. The trial protocols were reviewed and approved by regional ethics review boards at all participating centres and complied with the ethical principles of the Declaration of Helsinki. The protocol for the present *post hoc* analysis was approved by the Swedish Ethical Review Authority (registration number: 2019–05498).

### Clinical definitions

Mucocutaneous SLE disease activity was evaluated using the mucocutaneous domain of the classic version of the BILAG (mcBILAG) index [21] and the mucocutaneous organ system section of SLEDAI 2000 (mcSLEDAI-2K; alopecia, mucous membrane lesions and rash) [22]. Assessments of both BILAG and SLEDAI-2K were performed at every visit, i.e. every fourth week.

Improvement by mcBILAG was defined as a change in mcBILAG from a baseline score A to a score B, C or D, or a change from a baseline score B to a score C or D. Improvement by mcSLEDAI-2K was defined as a ≥ 2-point reduction in the total mcSLEDAI-2K score compared with baseline.

Sustained mcBILAG and sustained mcSLEDAI-2K improvement were defined as improvement from baseline persisting for at least two consecutive visits and being maintained through week 52.

Flares by mcBILAG were defined as a switch to mcBILAG A in patients with mcBILAG B, C, D or E at baseline, or a switch to mcBILAG B in patients with mcBILAG C, D or E at baseline.

For subgroup analyses, patients were stratified according to the presence or absence of the following features at the baseline assessment, proxies for high disease activity: (i) SLEDAI-2K ≥10, (ii) anti-dsDNA antibody positivity (≥30 IU/ml), (iii) complement consumption (C3 < 90.0 mg/dl in all trials, or C4 < 16.0 mg/dl in BLISS-52 and BLISS-76 or <10.0 mg/dl in BLISS-NEA, BLISS-SC, and EMBRACE) and (iv) glucocorticoid use at a prednisone equivalent dose of >7.5 mg/day.

### Statistical analysis

Results from descriptive statistics are reported as numbers (percentage) or means (standard deviation) for normally distributed data or medians (interquartile range) for non-normal distributions. For comparisons, the non-parametrical Mann–Whitney *U* test was used for continuous variables and the Pearson’s chi-squared (*χ*^2^) test for dichotomous variables, as appropriate. For comparisons between belimumab and placebo, placebo-treated patients in the respective trials formed the comparator groups.

Results from logistic regression models are presented as the odds ratio (OR), the 95% confidence interval (CI), and the *P*-value. Proportional hazards (Cox) regression analysis was used to assess belimumab efficacy in inducing sustained improvement and in preventing flares over time. These data are presented as the hazard ratio (HR), the 95% CI, and the *P*-value. Logistic and Cox regression analyses were adjusted for trial variance, with BLISS-52 and BLISS-76 pooled as a reference.

Differences yielding *P* values < 0.05 were deemed statistically significant. Analyses were performed and illustrations were developed using R version 4.2.1 (R Foundation for Statistical Computing, Vienna, Austria).

## Results

Baseline patient characteristics are shown in Table 1; no substantial differences were observed between the treatment groups.

**Table 1.** Baseline patient characteristics

	Placebo (*N* = 1217)	Belimumab (*N* = 1869)	All (*N* = 3086)
**Demographics**			
Age (years); mean (s.d.)	37.4 (12.0)	36.7 (11.4)	37.0 (11.6)
Female sex; *n* (%)	1144 (94.0)	1769 (94.6)	2913 (94.4)
Ethnicity; *n* (%)			
Asian	405 (33.3)	698 (37.3)	1103 (35.7)
Black/African American	229 (18.8)	403 (21.6)	632 (20.5)
Indigenous American	147 (12.1)	172 (9.2)	319 (10.3)
White/Caucasian	436 (35.8)	596 (31.9)	1032 (33.4)
**Clinical data at baseline**			
SLE duration (years); mean (s.d.)	6.6 (6.5)	6.3 (6.3)	6.4 (6.4)
PGA; mean (s.d.)	1.5 (0.5)	1.5 (0.5)	1.5 (0.5)
Anti-dsDNA (+); *n* (%)	849 (69.8)	1352 (72.3)	2201 (71.3)
Low C3 and/or low C4; *n* (%)	689 (56.6)	1073 (57.4)	1762 (57.1)
anti-dsDNA (+) and low C3 and/or low C4; *n* (%)	580 (47.7)	935 (50.0)	1515 (49.1)
mcBILAG score; *n* (%)			
A	55 (4.5)	77 (4.1)	132 (4.3)
B	697 (57.3)	1010 (54.0)	1707 (55.3)
C	338 (27.8)	558 (29.9)	896 (29.0)
D	13 (1.1)	27 (1.4)	40 (1.3)
E	114 (9.4)	197 (10.5)	311 (10.1)
SLEDAI-2K score; mean (s.d.)	10.3 (3.6)	10.5 (3.6)	10.4 (3.6)
SLEDAI-2K <10; *n* (%)	500 (41.1)	757 (40.5)	1257 (40.7)
SLEDAI-2K ≥10; *n* (%)	717 (58.9)	1112 (59.5)	1829 (59.3)
mcSLEDAI-2K score; *n* (%)			
0	178 (14.6)	284 (15.2)	462 (15.0)
2	403 (33.1)	610 (32.6)	1013 (32.8)
4	475 (39.0)	744 (39.8)	1219 (39.5)
6	161 (13.2)	231 (12.4)	392 (12.7)
mcSLEDAI-2K descriptors;			
Alopecia; *n* (%)	728 (59.8)	1133 (60.6)	1861 (60.3)
Mucosal ulcers; *n* (%)	314 (25.8)	501 (26.8)	815 (26.4)
Rash; *n* (%)	794 (65.2)	1157 (61.9)	1951 (63.2)
mcSLEDAI-2K score; mean (s.d.)	3.0 (1.8)	3.0 (1.8)	3.0 (1.8)
**Medications**			
Prednisone equivalent dose at baseline; mean (s.d.)	12.2 (9.6)	12.3 (9.7)	12.3 (9.7)
Prednisone eq. 0 mg; *n* (%)	138 (11.3)	221 (11.8)	359 (11.6)
Prednisone eq. ≤7.5 mg; *n* (%)	314 (25.8)	453 (24.2)	767 (24.9)
Prednisone eq. >7.5 mg; *n* (%)	765 (62.9)	1195 (63.9)	1960 (63.5)
AMA at baseline; *n* (%)	851 (69.9)	1301 (69.6)	2152 (69.7)
IS at baseline; *n* (%)	654 (53.7)	981 (52.5)	1635 (53.0)
GC, AMA, IS at baseline; *n* (%)	363 (29.8)	588 (31.5)	951 (30.8)

AMA: antimalarial agents; Anti-dsDNA: anti double-stranded DNA antibodies: eq.: equivalent; GC: glucocorticoids; IS: immunosuppressants; mcBILAG: mucocutaneous Bristish Isles Lupus Assessment Group; mcSLEDAI-2K: mucocutaneous SLEDAI 2000; PGA: physician global assessment; SLEDAI-2K: SLEDAI 2000.

### mcBILAG-based improvement

At baseline, 132 patients (4.3%) had a mcBILAG A and 1707 (55.3%) a mcBILAG B. Upon randomization, 1087 patients received the approved dose of belimumab and 752 received placebo on top of ST.

**Figure 1.**
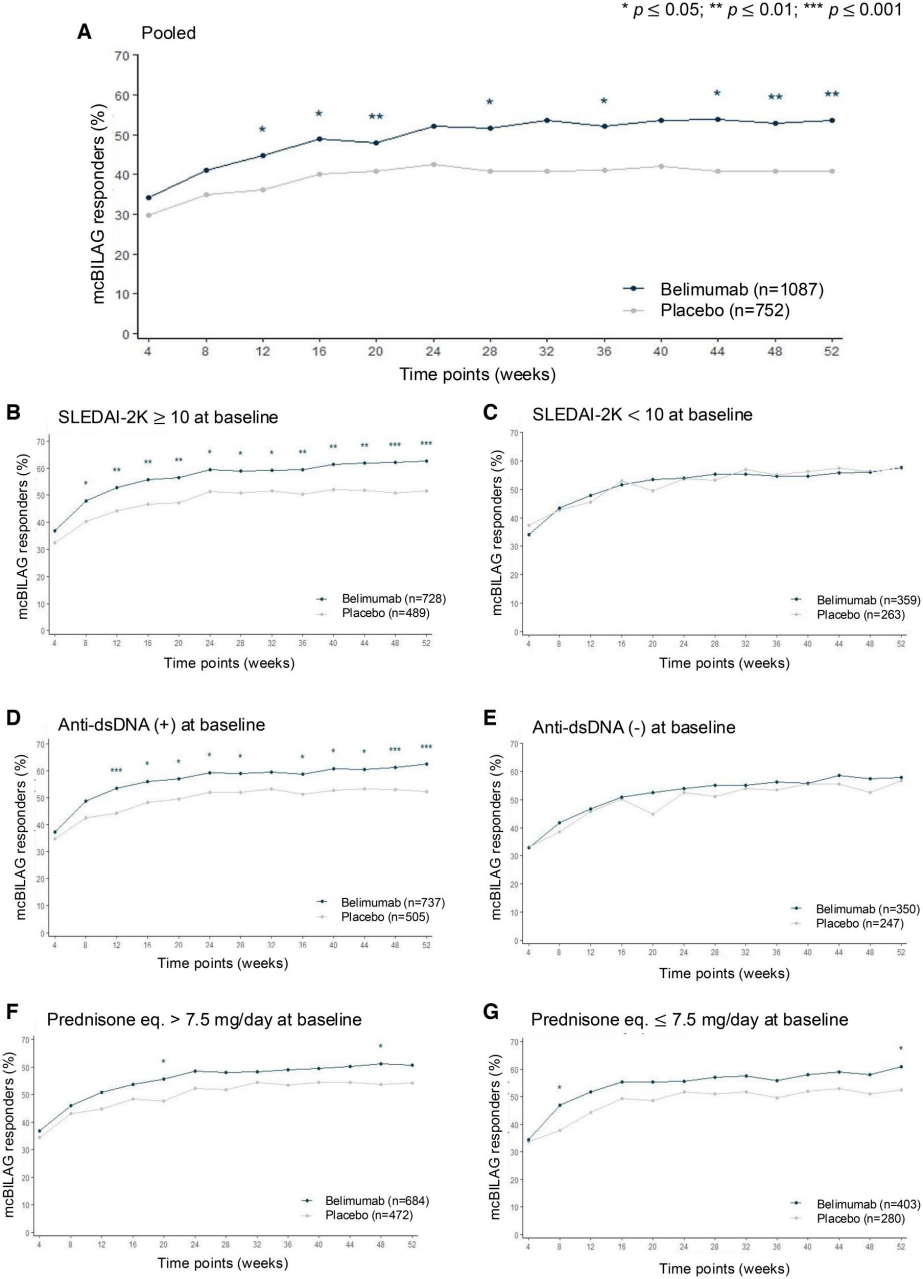
mcBILAG improvement from week 4 through week 52 in SLE patients treated with belimumab vs. placebo. (A) Pooled belimumab RCT population. (B) SLE patients with SLEDAI-2K score ≥10 at baseline. (C) SLE patients with SLEDAI-2K <10 at baseline. (D) SLE patients with positive anti-dsDNA levels at baseline. (E) SLE patients negative for anti-dsDNA at baseline. (F) SLE patients with prednisone (or equivalent) dose >7.5 mg/day at baseline. (G) SLE patients with prednisone (or equivalent) dose ≤7.5 mg/day at baseline. Anti-dsDNA: anti double-stranded DNA antibodies; eq.: equivalent; mcBILAG: mucocutaneous BILAG; RCT: randomized controlled trial; SLE: systemic lupus erythematosus; SLEDAI-2K: SLEDAI 2000

A benefit from belimumab regarding mcBILAG-based improvement was first observed at week 12 (OR: 1.28; 95% CI: 1.06–1.54; *P* = 0.012). At week 52, mcBILAG-based improvement was reached by 662 (60.9%) patients on belimumab vs. 403 (53.6%) on placebo (OR: 1.29; 95% CI: 1.07–1.57; *P* = 0.008; Supplementary Table S1, available at *Rheumatology* online; Fig. 1A).

In patients with baseline SLEDAI-2K scores ≥10 (*N* = 1217; 728 on belimumab, 489 on placebo), a difference in favour of belimumab was seen from week 8 (OR: 1.33; 95% CI: 1.05–1.68; *P* = 0.017). At week 52, mcBILAG-based improvement was reached by 455 (62.5%) patients on belimumab vs. 252 (51.5%) on placebo (OR: 1.49; 95% CI: 1.18–1.89; *P* = 0.001; Supplementary Table S2, available at *Rheumatology* online; Fig. 1B). In patients with baseline SLEDAI-2K scores <10 (*N* = 622, 359 on belimumab, 263 on placebo), 207 (57.7%) patients on belimumab reached mcBILAG-based improvement at week 52 vs. 151 (57.4%) on placebo (OR: 1.00; 95% CI: 0.72–1.39; *P* = 0.977; Supplementary Table S3, available at *Rheumatology* online; Fig. 1C).

In patients with positive levels of anti-dsDNA antibodies at baseline (*N* = 1242; 737 on belimumab, 505 on placebo), belimumab benefit was first seen at week 12 (OR: 1.45; 95% CI: 1.15–1.83; *P* = 0.001). At week 52, mcBILAG-based improvement was reached by 459 (62.3%) patients on belimumab vs. 263 (52.1%) on placebo (OR: 1.44; 95% CI: 1.14–1.82; *P* = 0.002; Supplementary Table S4, available at *Rheumatology* online; Fig. 1D). In patients with negative baseline anti-dsDNA levels (*N* = 597; 350 on belimumab, 247 on placebo), the week-52 OR was 1.03 (95% CI: 0.73–1.44; *P* = 0.872; Supplementary Table S5, available at *Rheumatology* online; Fig. 1E). Similar patterns were seen for patients with anti-dsDNA positivity and/or low complement levels at baseline (Supplementary Table S6, available at *Rheumatology* online) and those with negative anti-dsDNA levels and normal/high complement levels (Supplementary Table S7, available at *Rheumatology* online).

In patients with a baseline prednisone equivalent dose >7.5 mg/day (*N* = 1156; 684 on belimumab, 472 on placebo), belimumab benefit was seen at week 20 (OR: 1.34; 95% CI: 1.05–1.70; *P* = 0.017) and week 48 (OR: 1.31; 95% CI: 1.03–1.67; *P* = 0.027; Supplementary Table S8, available at *Rheumatology* online; Fig. 1F). In patients with a baseline prednisone equivalent dose ≤7.5 mg/day (*N* = 683; 403 on belimumab, 280 on placebo), a difference between the groups in favour of belimumab was seen at week 4 (OR: 1.41; 95% CI: 1.02–1.94; *P* = 0.036) and week 52 (OR: 1.42; 95% CI: 1.03–1.94; *P* = 0.032; Supplementary Table S9, available at *Rheumatology* online; Fig. 1G).

### Sustained mcBILAG-based improvement

**Figure 2.**
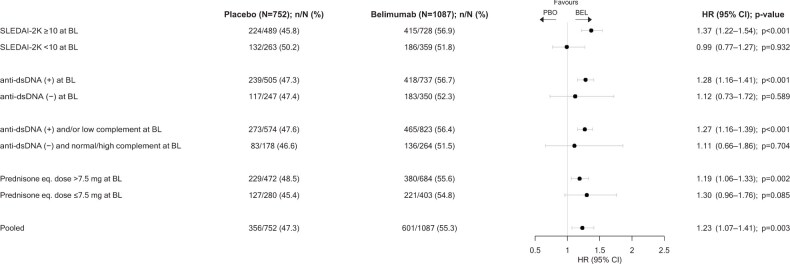
Sustained mcBILAG improvement through week 52 in SLE patients treated with belimumab vs. placebo. Plotted are results from the pooled belimumab RCT population and patient subgroups of interest. Anti-dsDNA: anti double-stranded DNA antibodies; BEL: belimumab; BL: baseline; eq.: equivalent; HR: hazard ratio; mcBILAG: mucocutaneous BILAG; PBO: placebo; RCT: randomized controlled trial; SLEDAI-2K: SLEDAI 2000

A total of 601 (55.3%) patients on belimumab vs. 356 (47.3%) on placebo demonstrated sustained mcBILAG-based improvement through week 52 (HR: 1.23; 95% CI: 1.07–1.41; *P* = 0.003), attained after a mean time of 139 days and 135 days, respectively (Fig. 2).

In patients with baseline SLEDAI-2K scores ≥10, 415 (56.9%) on belimumab vs. 224 (45.8%) on placebo attained sustained mcBILAG-based improvement through week 52 (HR: 1.37; 95% CI: 1.22–1.54; *P* < 0.001) after a mean time of 136 days and 141 days, respectively. In patients with baseline SLEDAI-2K scores <10, 186 (51.8%) on belimumab vs. 132 (50.2%) on placebo attained sustained mcBILAG-based improvement through week 52 (HR: 0.99; 95% CI: 0.77–1.27; *P* = 0.932) after a mean time of 148 days and 125 days, respectively, with no significant difference between the groups.

In patients with positive anti-dsDNA levels at baseline, 418 (56.7%) on belimumab vs. 239 (47.3%) on placebo attained sustained mcBILAG-based improvement through week 52 (HR: 1.28; 95% CI: 1.16–1.41; *P* < 0.001) after a mean time of 135 days and 132 days, respectively. In patients with negative anti-dsDNA levels, 183 (52.3%) on belimumab vs. 117 (47.4%) on placebo attained sustained mcBILAG-based improvement through week 52 (HR: 1.12; 95% CI: 0.73–1.72; *P* = 0.589) after a mean time of 150 days and 141 days, respectively.

In patients with a baseline prednisone equivalent dose >7.5 mg/day, 380 (55.6%) on belimumab vs. 229 (48.5%) on placebo demonstrated a sustained mcBILAG-based improvement through week 52 (HR: 1.19; 95% CI: 1.06–1.33; *P* = 0.002) after a mean time of 138 days and 130 days, respectively. In patients with a baseline prednisone equivalent dose ≤7.5 mg/day, 221 (54.8%) on belimumab vs. 127 (45.4%) on placebo demonstrated a sustained mcBILAG-based improvement through week 52 (HR: 1.30; 95% CI: 0.96–1.76; *P* = 0.085) after a mean time of 142 days and 144 days, respectively.

### mcSLEDAI-2K-based improvement

At baseline, the mean mcSLEDAI-2K score was 3.0; 462 patients (15.0%) had a mcSLEDAI-2K = 0 and were therefore excluded from this analysis (284 on belimumab, 178 on placebo), 1013 (32.8%) patients had mcSLEDAI-2K = 2 (610 on belimumab, 403 on placebo), 1219 (39.5%) had mcSLEDAI-2K = 4 (744 on belimumab, 475 on placebo), and 392 (12.7%) had mcSLEDAI-2K = 6 (231 on belimumab, 161 on placebo). Specifically, 1861 (60.3%) patients had alopecia, 815 (26.4%) had mucous membrane lesions, and 1951 (63.2%) had rash.

**Figure 3.**
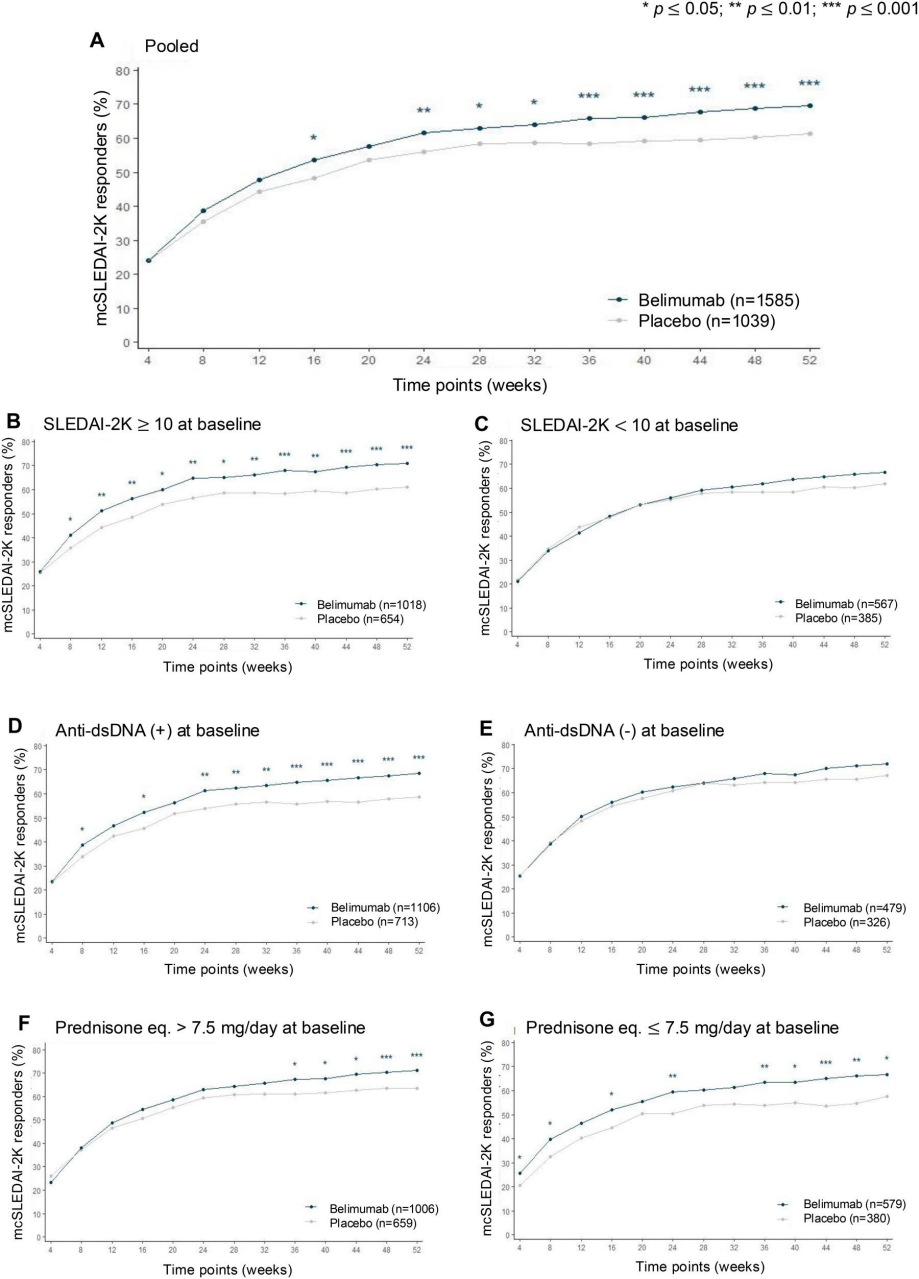
mcSLEDAI-2K improvement from week 4 through week 52 in SLE patients treated with belimumab vs. placebo. (A) Pooled belimumab RCT population. (B) SLE patients with SLEDAI-2K score ≥10 at baseline. (C) SLE patients with SLEDAI-2K < 10 at baseline. (D) SLE patients with positive anti-dsDNA levels at baseline. (E) SLE patients negative for anti-dsDNA at baseline. (F) SLE patients with prednisone (or equivalent) dose >7.5 mg/day at baseline. (G) SLE patients with prednisone (or equivalent) dose ≤7.5 mg/day at baseline. Anti-dsDNA: anti double-stranded DNA antibodies; eq.: equivalent; mcSLEDAI-2K: mucocutaneous SLEDAI 2000; RCT: randomized controlled trial; SLEDAI-2K: SLEDAI 2000

A benefit from belimumab regarding mcSLEDAI-2K-based improvement was first observed at week 16 (OR: 1.20; 95% CI: 1.02–1.41; *P* = 0.025). At week 52, mcSLEDAI-2K-based improvement was reached by 1102 (69.5%) patients on belimumab vs. 638 (61.4%) on placebo (OR: 1.37; 95% CI: 1.16–1.62; *P* < 0.001; Supplementary Table S10, available at *Rheumatology* online; Fig. 3A).

In patients with a baseline SLEDAI-2K score ≥10 (*N* = 1672; 1018 on belimumab, 654 on placebo), a difference between the groups was seen as early as at week 8 (OR: 1.28; 95% CI: 1.04–1.57; *P* = 0.019). At week 52, mcSLEDAI-2K-based improvement was attained by 723 (71.0%) patients on belimumab vs. 400 (61.2%) on placebo (OR: 1.49; 95% CI: 1.20–1.84; *P* < 0.001; Supplementary Table S11, available at *Rheumatology* online; Fig. 3B). In patients with baseline SLEDAI-2K scores <10 (*N* = 952; 567 on belimumab, 385 on placebo), 379 (66.8%) patients on belimumab reached mcSLEDAI-2K-based improvement at week 52 vs. 238 (61.8%) on placebo (OR: 1.2; 95% CI: 0.91–1.58; *P* = 0.198; Supplementary Table S12, available at *Rheumatology* online; Fig. 3C).

In patients with positive anti-dsDNA levels at baseline (*N* = 1819; 1106 on belimumab, 713 on placebo), a benefit from belimumab was seen as early as at week 8 (OR: 1.27; 95% CI: 1.04–1.55; *P* = 0.020). At week 52, mcSLEDAI-2K-based improvement was reached by 757 (68.4%) patients on belimumab vs. 419 (58.8%) on placebo (OR: 1.47; 95% CI: 1.20–1.79; *P* < 0.001; Supplementary Table S13, available at *Rheumatology* online; Fig. 3D). In patients with absence of anti-dsDNA antibodies at baseline (*N* = 805; 479 on belimumab, 326 on placebo), 345 (72%) patients on belimumab reached mcSLEDAI-2K-based improvement at week 52 vs. 219 (67.2%) on placebo (OR: 1.16; 95% CI: 0.85–1.58; *P* = 0.360; Supplementary Table S14, available at *Rheumatology* online; Fig. 3E). At weeks 12 and 52, outcomes for patients with anti-dsDNA positivity and/or low complement were analogous to those observed in the anti-dsDNA positivity group (Supplementary Table S15, available at *Rheumatology* online). Conversely, patients with negative anti-dsDNA levels and normal/high complement levels exhibited results consistent with those seen in patients with negative anti-dsDNA levels (Supplementary Table S16, available at *Rheumatology* online).

In patients with a baseline prednisone equivalent dose >7.5 mg/day (*N* = 1665; 1006 on belimumab, 659 on placebo), a benefit from belimumab was first seen at week 36 (OR: 1.29; 95% CI: 1.04–1.58; *P* = 0.018). At week 52, mcSLEDAI-2K-based improvement was reached by 715 (71.1%) patients on belimumab vs. 419 (63.6%) on placebo (OR: 1.36; 95% CI: 1.10–1.68; *P* = 0.004; Supplementary Table S17, available at *Rheumatology* online; Fig. 3F). In patients with a baseline prednisone equivalent dose ≤7.5 mg/day (*N* = 959; 579 on belimumab, 380 on placebo), a difference between the groups was seen as early as at week 4 (OR: 1.38; 95% CI: 1.01–1.90; *P* = 0.045). At week 52, mcSLEDAI-2K-based improvement was reached by 387 (66.8%) patients on belimumab vs. 219 (57.6%) on placebo (OR: 1.42; 95% CI: 1.08–1.86; *P* = 0.013; Supplementary Table S18, available at *Rheumatology* online; Fig. 3G).

### Sustained mcSLEDAI-2K-based improvement

A total of 1060 (66.9%) patients on belimumab vs. 605 (58.2%) on placebo demonstrated sustained mcSLEDAI-2K-based improvement through week 52 (HR: 1.24; 95% CI: 1.17–1.31; *P* < 0.001), reached after a mean time of 115 days in both groups.

In patients with baseline SLEDAI-2K scores ≥10, 697 (68.4%) on belimumab vs. 378 (57.8%) on placebo attained sustained mcSLEDAI-2K-based improvement through week 52 (HR: 1.32; 95% CI: 1.17–1.50; *P* < 0.001) after a mean time of 108 and 113 days, respectively. In patients with baseline SLEDAI-2K scores <10, 363 (64.0%) on belimumab vs. 227 (59.0%) on placebo attained sustained mcSLEDAI-2K-based improvement (HR: 1.11; 95% CI: 0.99–1.24; *P* = 0.087) after a mean time of 128 days and 119 days, respectively.

In patients with anti-dsDNA positivity at baseline, 724 (65.5%) on belimumab vs. 398 (55.8%) on placebo demonstrated sustained mcSLEDAI-2K-based improvement (HR: 1.29; 95% CI: 1.23–1.35; *P* < 0.001) after a mean time of 114 and 118 days, respectively. In patients with negative anti-dsDNA antibody levels, 336 (70.1%) on belimumab vs. 207 (63.5%) on placebo demonstrated sustained mcSLEDAI-2K-based improvement (HR: 1.15; 95% CI: 0.99–1.34; *P* = 0.069) after a mean time of 116 and 110 days, respectively.

**Figure 4.**
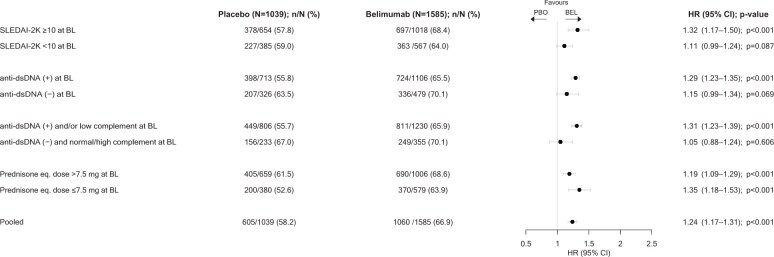
Sustained mcSLEDAI-2K improvement through week 52 in SLE patients treated with belimumab vs. placebo. Plotted are results from the pooled belimumab RCT population and patient subgroups of interest. Anti-dsDNA: anti double-stranded DNA antibodies; BEL: belimumab; BL: baseline; eq.: equivalent; HR: hazard ratio; mcSLEDAI-2K: mucocutaneous SLEDAI 2000; PBO: placebo; RCT: randomized controlled trial; SLEDAI-2K: SLEDAI 2000

In patients with baseline prednisone equivalent dose >7.5 mg/day, 690 (68.6%) on belimumab vs. 405 (61.5%) on placebo demonstrated sustained mcSLEDAI-2K-based improvement (HR: 1.19; 95% CI: 1.09–1.29; *P* < 0.001) reached after a mean time of 112 and 113 days, respectively. In patients with baseline prednisone equivalent dose ≤7.5 mg/day, 370 (63.9%) on belimumab vs. 200 (52.6%) on placebo displayed sustained mcSLEDAI-2K-based improvement (HR: 1.35; 95% CI: 1.18–1.53; *P* < 0.001) reached after a mean time of 119 days and 121 days, respectively (Fig. 4).

### mcBILAG flares

At baseline, 1707 (55.3%) patients had mcBILAG B, 896 (29.0%) had mcBILAG C, 40 (1.3%) had mcBILAG D, and 311 (10.1%) had mcBILAG E. Upon randomization, 1792 received add-on belimumab, whereas 1162 received placebo.

During the study period, 193 (10.8%) patients on belimumab experienced at least one mcBILAG-based flare with the first flare being documented after a mean time of 128 days vs. 149 (12.8%) patients on placebo with the first flare being documented after a mean time of 136 days (HR: 0.83; 95% CI: 0.64–1.09; *P* = 0.177).

In patients with baseline SLEDAI-2K scores ≥10 (*N* = 1736; 1053 on belimumab, 683 on placebo), 103 (9.8%) patients on belimumab experienced at least one mcBILAG-based flare with the first flare being documented after a mean time of 128 days vs. 92 (13.5%) patients on placebo with the first flare being documented after a mean time of 131 days (HR: 0.71; 95% CI: 0.51–1.00; *P* = 0.050). In patients with baseline SLEDAI-2K scores <10 (*N* = 1218; 739 on belimumab, 479 on placebo), 90 (12.2%) patients on belimumab experienced at least one mcBILAG-based flare with the first flare being documented after a mean time of 129 days vs. 57 (11.9%) patients on placebo with the first flare being documented after a mean time of 144 days (HR: 1.03; 95% CI: 0.89–1.20; *P* = 0.674).

In patients with anti-dsDNA antibody positivity at baseline (*N* = 2102; 1296 on belimumab, 806 on placebo), 138 (10.7%) patients on belimumab experienced at least one mcBILAG-based flare with the first flare being documented after a mean time of 128 days vs. 120 (14.9%) on placebo with the first flare being documented after a mean time of 130 days (HR: 0.70; 95% CI: 0.50–0.98; *P* = 0.035). In patients with absence of anti-dsDNA antibodies at baseline (*N* = 852; 496 on belimumab, 356 on placebo), 55 (11.1%) patients on belimumab experienced at least one mcBILAG-based flare with the first flare being documented after a mean time of 130 days vs. 29 (8.1%) on placebo with the first flare being documented after a mean time of 162 days (HR: 1.39; 95% CI: 0.99–1.96; *P* = 0.055).

**Figure 5.**
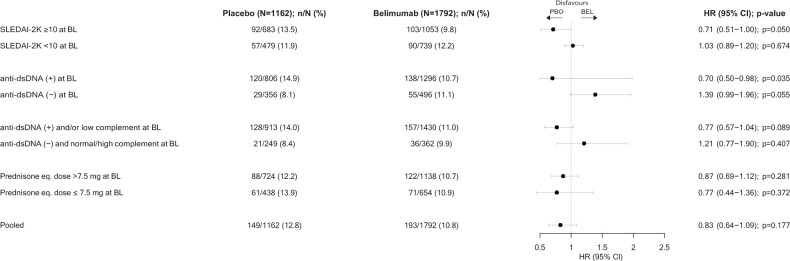
mcBILAG flares through week 52 in SLE patients treated with belimumab vs. placebo Plotted are results from the pooled belimumab RCT population and patient subgroups of interest. Anti-dsDNA: anti double-stranded DNA antibodies; BEL: belimumab; BL: baseline; eq.: equivalent; HR: hazard ratio; mcBILAG: mucocutaneous BILAG; PBO: placebo; RCT: randomized controlled trial; SLEDAI-2K: SLEDAI 2000.

In patients with a baseline prednisone equivalent dose >7.5 mg/day (*N* = 1862; 1138 on belimumab, 724 on placebo), 122 (10.7%) patients on belimumab experienced at least one mcBILAG-based flare with the first flare being documented after a mean time of 136 days vs. 88 (12.2%) on placebo with the first flare being documented after a mean time of 133 days (HR: 0.87; 95% CI: 0.69–1.12; *P* = 0.281). In patients with baseline prednisone equivalent dose ≤7.5 mg/day (*N* = 1092; 654 on belimumab, 438 on placebo), 71 (10.9%) patients on belimumab experienced at least one mcBILAG-based flare with the first flare being documented after a mean time of 116 days vs. 61 (13.9%) on placebo with the first flare being documented after a mean time of 140 days (HR: 0.77; 95% CI: 0.44–1.36; *P* = 0.372; Fig. 5).

## Discussion

Current treatment recommendations for SLE are mainly based on organ involvement [18]. Mucocutaneous affliction is one of the most common manifestations of the disease [23]. To inform future targeted treatment strategies, we aimed to determine the efficacy of belimumab in mucocutaneous SLE in the largest to date placebo-controlled clinical trial population. We demonstrated superiority of belimumab over placebo in inducing not only improvement and sustained improvement in mucocutaneous SLE, but also prevention of mucocutaneous SLE flares. These results were even more prominent in subgroups of patients denoting high disease activity at baseline. Considering constant advancements in SLE pharmacotherapy and enrichments of the therapeutic armamentarium, our findings constitute a foundation for comparisons across drugs employed to treat SLE with regard to their efficacy in mucocutaneous SLE.

We assessed treatment response using two different disease activity measures, mcBILAG [21] and mcSLEDAI-2K [22]. Statistically significant differences regarding improvement in favour of belimumab were seen from week 12 when improvement was defined using mcBILAG and from week 16 when mcSLEDAI-2K was used. The earlier separation between belimumab and placebo with mcBILAG could be explained by the intrinsic differences in these two measures; mcBILAG can capture improvement or worsening in persisting mucocutaneous manifestations, whereas mcSLEDAI-2K is a binary scoring system for each descriptor that indicates presence or absence of a clinical symptom and is not designed to denote improvement or worsening. Thus, mcBILAG may be anticipated to be more sensitive to improvements that do not change the binary status of a manifestation.

This is not the first study investigating the effect of belimumab on mucocutaneous SLE. A previous *post hoc* analysis of BLISS-52 and BLISS-76 by Manzi *et al.* assessed the efficacy of belimumab in distinct organ domains, including mucocutaneous manifestations of SLE, and demonstrated improvement from baseline to week 52 [23]. Real-world data, although with smaller cohorts and without a control group [24–31], have also demonstrated belimumab efficacy using mcSLEDAI-2K and the mcSLE-specific Cutaneous Lupus erythematosus disease Area and Severity Index (CLASI) [32]. In the Italian BeRLiSS cohort [27], belimumab was shown to contribute to improvement in the mucocutaneous domain, i.e. low disease activity and remission in 32.4% and 16.2% of patients, respectively, after 6 months, which was the time for the first follow-up evaluation in that study, with the percentage increasing with time until the end of follow-up at month 36. In that study, SLEDAI-2K or anti-dsDNA antibodies were not associated with improvement in the mucocutaneous domain. Another Italian study demonstrated the efficacy of belimumab in reducing CLASI activity as well as preventing disease flares at 12, 18 and 24 months compared with up to 24 months before belimumab commencement [24]. However, flares were defined using the SELENA-SLEDAI Flare Index, expressed as number of flares per 100 patients, and were not limited to the mucocutaneous domain. The OBSErve USA [25], Spain [31], Germany [26], Argentina [33], Canada [34], and Switzerland [35], as well as a study from a Swedish clinical setting [28], demonstrated real-world evidence of the efficacy of belimumab in inducing improvement in the mucocutaneous domain at 6 months, with OBSErve USA [25] and Argentina [33] showing responses that were maintained through month 24 from baseline.

With this study, we add more information by studying a larger and ethnically more heterogeneous population from five phase III trials, with the advantage of a placebo group that allowed us to perform direct comparisons to unveil the added value of belimumab on top of non-biological ST. With the premise that maintenance of a favourable effect upon its induction is crucial towards prevention of organ damage accrual, we also investigated sustained improvement; belimumab induced improvements that were sustained for at least two consecutive visits and maintained through week 52 to a greater extent than placebo.

Limitations of this study include its *post hoc* nature. The trials were not designed to specifically assess the efficacy of belimumab across organ systems. The SLE population was restricted to the trial-specific eligibility criteria, which infers a selection bias. Therefore, the outcomes may not be fully generalizable to real-world SLE populations. Additionally, CLASI, which would have allowed a more granular characterization of mucocutaneous involvement, was not used in the trials, and mucocutaneous involvement was analysed as a pooled manifestation, preventing differentiation between specific subsets of cutaneous involvement (acute, subacute, chronic) or isolated mucosal involvement. However, the patient population was large, ancestrally heterogeneous, and with a consistent and meticulous follow-up of 52 weeks across the trials, the similar design of which facilitated data pooling and statistical empowerment.

In summary, the present large integrative *post hoc* analysis of five phase III clinical trials demonstrated superiority of belimumab over placebo on top of background non-biological ST in inducing improvement in mucocutaneous manifestations of SLE from as early as week 12 with mcBILAG and week 16 with mcSLEDAI-2K, as well as sustained and maintained improvement through week 52 assessed with both mcBILAG and mcSLEDAI-2K. The benefit from belimumab in inducing improvements in the mucocutaneous domain was more prominent in patients with high disease activity and in serologically active patients at baseline. Our study also demonstrated greater benefit conferred from belimumab vs. placebo in preventing mucocutaneous flares in patients with SLE who were serologically active at baseline. These findings justify the use of belimumab for treating mucocutaneous manifestations of SLE, with improvements expected from three months and resolution of symptoms from four months upon treatment commencement.

## Supplementary Material

keaf145_Supplementary_Data

## Data Availability

The datasets used and analysed during the current study are available upon request through the CSDR consortium.
