# Supplementary material for: Belimumab efficacy in mucocutaneous manifestations of systemic lupus erythematosus: a large post hoc analysis of five phase III clinical trials
Source: Rheumatology (Oxford). 2025 Mar 14;64(7):4257–66. doi: 10.1093/rheumatology/keaf145 (PMC12212913; doi:10.1093/rheumatology/keaf145)
Supplement: keaf145_Supplementary_Data [file keaf145_supplementary_data.docx]

**SUPPLEMENTARY MATERIAL**

TABLE OF CONTENTS

[Supplementary Table S1. mcBILAG improvement from week 4 through week 52 in patients with SLE treated with belimumab versus placebo in the pooled belimumab RCT population. 4](#_Toc185192813)

[Supplementary Table S2. mcBILAG improvement from week 4 through week 52 in patients with SLEDAI-2K ≥ 10 at baseline treated with belimumab versus placebo. 5](#_Toc185192814)

[Supplementary Table S3. mcBILAG improvement from week 4 through week 52 in patients with SLEDAI-2K < 10 at baseline treated with belimumab versus placebo. 6](#_Toc185192815)

[Supplementary Table S4. mcBILAG improvement from week 4 through week 52 in patients with positive anti-dsDNA levels at baseline treated with belimumab versus placebo. 7](#_Toc185192816)

[Supplementary Table S5. mcBILAG improvement from week 4 through week 52 in patients with negative anti-dsDNA levels at baseline treated with belimumab versus placebo. 8](#_Toc185192817)

[Supplementary Table S6. mcBILAG improvement from week 4 through week 52 in patients with positive anti-dsDNA levels and/or low complement levels at baseline treated with belimumab versus placebo. 9](#_Toc185192818)

[Supplementary Table S7. mcBILAG improvement from week 4 through week 52 in patients with negative anti-dsDNA levels and normal/high complement levels at baseline treated with belimumab versus placebo. 10](#_Toc185192819)

[Supplementary Table S8. mcBILAG improvement from week 4 through week 52 in patients receiving prednisone equivalent dose > 7.5 mg/day at baseline treated with belimumab versus placebo. 11](#_Toc185192820)

[Supplementary Table S9. mcBILAG improvement from week 4 through week 52 in patients receiving prednisone equivalent dose ≤ 7.5 mg/day at baseline treated with belimumab versus placebo. 12](#_Toc185192821)

[Supplementary Table S10. mcSLEDAI-2K improvement from week 4 through week 52 in patients with SLE treated with belimumab versus placebo in the pooled belimumab RCT population. 13](#_Toc185192822)

[Supplementary Table S11. mcSLEDAI-2K improvement from week 4 through week 52 in patients with SLEDAI-2K ≥ 10 at baseline treated with belimumab versus placebo. 14](#_Toc185192823)

[Supplementary Table S12. mcSLEDAI-2K improvement from week 4 through week 52 in patients with SLEDAI-2K < 10 at baseline treated with belimumab versus placebo. 15](#_Toc185192824)

[Supplementary Table S13. mcSLEDAI-2K improvement from week 4 through week 52 in patients with positive anti-dsDNA levels at baseline treated with belimumab versus placebo. 16](#_Toc185192825)

[Supplementary Table S14. mcSLEDAI-2K improvement from week 4 through week 52 in patients with negative anti-dsDNA levels at baseline treated with belimumab versus placebo. 17](#_Toc185192826)

[Supplementary Table S15. mcSLEDAI-2K improvement from week 4 through week 52 in patients with positive anti-dsDNA levels and/or low complement levels at baseline treated with belimumab versus placebo. 18](#_Toc185192827)

[Supplementary Table S16. mcSLEDAI-2K improvement from week 4 through week 52 in patients with negative anti-dsDNA levels and normal/high complement levels at baseline treated with belimumab versus placebo. 19](#_Toc185192828)

[Supplementary Table S17. mcSLEDAI-2K improvement from week 4 through week 52 in patients receiving prednisone equivalent dose > 7.5 mg/day at baseline treated with belimumab versus placebo. 20](#_Toc185192829)

[Supplementary Table S18. mcSLEDAI-2K improvement from week 4 through week 52 in patients receiving prednisone equivalent dose ≤ 7.5 mg/day at baseline treated with belimumab versus placebo. 21](#_Toc185192830)

# **Supplementary Table S1.** mcBILAG improvement from week 4 through week 52 in patients with SLE treated with belimumab versus placebo in the pooled belimumab RCT population.

|  | BEL; n (%)  N=1087 | PBO; n (%)  N=752 | OR | 95% CI | *p* value |
| --- | --- | --- | --- | --- | --- |
| **mcBILAG** **improvement** | | | | | |
| mcBILAG at week 4 | 390 (35.9) | 257 (34.2) | 1.08 | 0.89–1.32 | 0.438 |
| mcBILAG at week 8 | 504 (46.4) | 309 (41.1) | 1.19 | 0.98–1.44 | 0.079 |
| mcBILAG at week 12 | 557 (51.2) | 336 (44.7) | 1.28 | 1.06–1.54 | **0.012** |
| mcBILAG at week 16 | 591 (54.4) | 367 (48.8) | 1.21 | 1.00–1.46 | **0.048** |
| mcBILAG at week 20 | 603 (55.5) | 361 (48.0) | 1.31 | 1.09–1.59 | **0.005** |
| mcBILAG at week 24 | 625 (57.5) | 392 (52.1) | 1.20 | 0.99–1.45 | 0.063 |
| mcBILAG at week 28 | 627 (57.7) | 388 (51.6) | 1.23 | 1.01–1.48 | **0.037** |
| mcBILAG at week 32 | 630 (58.0) | 402 (53.5) | 1.13 | 0.93–1.37 | 0.212 |
| mcBILAG at week 36 | 629 (57.9) | 391 (52.0) | 1.22 | 1.01–1.48 | **0.040** |
| mcBILAG at week 40 | 642 (59.1) | 403 (53.6) | 1.18 | 0.98–1.43 | 0.088 |
| mcBILAG at week 44 | 650 (59.8) | 405 (53.9) | 1.25 | 1.03–1.51 | **0.023** |
| mcBILAG at week 48 | 652 (60.0) | 397 (52.8) | 1.30 | 1.07–1.57 | **0.007** |
| mcBILAG at week 52 | 662 (60.9) | 403 (53.6) | 1.29 | 1.07–1.57 | **0.008** |

Results from logistic regression, adjusted for trial variance with BLISS-52 and BLISS-76 pooled as a reference, of mcBILAG improvement from week 4 through week 52 in patients with SLE treated with belimumab versus placebo in the pooled belimumab RCT population. Data are presented as number of events (percentage), odds ratio (OR), 95% confidence interval (CI), and *p* value. Statistically significant *p* values are in bold.

BEL: belimumab; mcBILAG: mucocutaneous British Isles Lupus Assessment Group; CI: confidence interval; NA: not applicable; OR: odds ratio; PBO: placebo, RCT: randomized controlled trial.

# **Supplementary Table S2.** mcBILAG improvement from week 4 through week 52 in patients with SLEDAI-2K ≥ 10 at baseline treated with belimumab versus placebo.

|  | BEL; n (%)  N=728 | PBO; n (%)  N=489 | OR | 95% CI | *p* value |
| --- | --- | --- | --- | --- | --- |
| **mcBILAG** **improvement** | | | | | |
| mcBILAG at week 4 | 268 (36.8) | 159 (32.5) | 1.26 | 0.99–1.62 | 0.064 |
| mcBILAG at week 8 | 348 (47.8) | 197 (40.3) | 1.33 | 1.05–1.68 | **0.017** |
| mcBILAG at week 12 | 385 (52.9) | 216 (44.2) | 1.42 | 1.12–1.79 | **0.003** |
| mcBILAG at week 16 | 406 (55.8) | 228 (46.6) | 1.42 | 1.12–1.79 | **0.003** |
| mcBILAG at week 20 | 411 (56.5) | 231 (47.2) | 1.42 | 1.13–1.80 | **0.003** |
| mcBILAG at week 24 | 432 (59.3) | 251 (51.3) | 1.34 | 1.05–1.70 | **0.014** |
| mcBILAG at week 28 | 428 (58.8) | 248 (50.7) | 1.33 | 1.05–1.68 | **0.017** |
| mcBILAG at week 32 | 431 (59.2) | 252 (51.5) | 1.28 | 1.01–1.62 | **0.041** |
| mcBILAG at week 36 | 433 (59.5) | 246 (50.3) | 1.40 | 1.10–1.76 | **0.005** |
| mcBILAG at week 40 | 446 (61.3) | 255 (52.1) | 1.37 | 1.08–1.73 | **0.009** |
| mcBILAG at week 44 | 450 (61.8) | 254 (51.9) | 1.45 | 1.15–1.84 | **0.002** |
| mcBILAG at week 48 | 451 (62.0) | 249 (50.9) | 1.51 | 1.19–1.91 | **0.001** |
| mcBILAG at week 52 | 455 (62.5) | 252 (51.5) | 1.49 | 1.18–1.89 | **0.001** |

Results from logistic regression, adjusted for trial variance with BLISS-52 and BLISS-76 pooled as a reference, of mcBILAG improvement from week 4 through week 52 in patients with SLEDAI-2K score ≥ 10 at baseline treated with belimumab versus placebo. Data are presented as number of events (percentage), odds ratio (OR), 95% confidence interval (CI), and *p* value. Statistically significant *p* values are in bold.

BEL: belimumab; mcBILAG: mucocutaneous British Isles Lupus Assessment Group; CI: confidence interval; NA: not applicable; OR: odds ratio; PBO: placebo; SLEDAI-2K: Systemic Lupus Erythematosus Disease Activity Index 2000.

# **Supplementary Table S3.** mcBILAG improvement from week 4 through week 52 in patients with SLEDAI-2K < 10 at baseline treated with belimumab versus placebo.

|  | BEL; n (%)  N=359 | PBO; n (%)  N=263 | OR | 95% CI | *p* value |
| --- | --- | --- | --- | --- | --- |
| **mcBILAG** **improvement** | | | | | |
| mcBILAG at week 4 | 122 (34.0) | 98 (37.3) | 0.80 | 0.57–1.13 | 0.204 |
| mcBILAG at week 8 | 156 (43.5) | 112 (42.6) | 0.93 | 0.66–1.30 | 0.662 |
| mcBILAG at week 12 | 172 (47.9) | 120 (45.6) | 1.03 | 0.74–1.43 | 0.850 |
| mcBILAG at week 16 | 185 (51.5) | 139 (52.9) | 0.89 | 0.64–1.23 | 0.483 |
| mcBILAG at week 20 | 192 (53.5) | 130 (49.4) | 1.11 | 0.80–1.54 | 0.539 |
| mcBILAG at week 24 | 193 (53.8) | 141 (53.6) | 0.96 | 0.69–1.33 | 0.789 |
| mcBILAG at week 28 | 199 (55.4) | 140 (53.2) | 1.04 | 0.75–1.45 | 0.810 |
| mcBILAG at week 32 | 199 (55.4) | 150 (57.0) | 0.89 | 0.64–1.24 | 0.507 |
| mcBILAG at week 36 | 196 (54.6) | 145 (55.1) | 0.95 | 0.68–1.32 | 0.747 |
| mcBILAG at week 40 | 196 (54.6) | 148 (56.3) | 0.90 | 0.65–1.25 | 0.517 |
| mcBILAG at week 44 | 200 (55.7) | 151 (57.4) | 0.94 | 0.67–1.30 | 0.700 |
| mcBILAG at week 48 | 201 (56.0) | 148 (56.3) | 0.98 | 0.71–1.37 | 0.924 |
| mcBILAG at week 52 | 207 (57.7) | 151 (57.4) | 1.00 | 0.72–1.39 | 0.977 |

Results from logistic regression, adjusted for trial variance with BLISS-52 and BLISS-76 pooled as a reference, of mcBILAG improvement from week 4 through week 52 in patients with SLEDAI-2K score < 10 at baseline treated with belimumab versus placebo. Data are presented as number of events (percentage), odds ratio (OR), 95% confidence interval (CI), and *p* value. Statistically significant *p* values are in bold.

BEL: belimumab; mcBILAG: mucocutaneous British Isles Lupus Assessment Group; CI: confidence interval; NA: not applicable; OR: odds ratio; PBO: placebo; SLEDAI-2K: Systemic Lupus Erythematosus Disease Activity Index 2000.

# **Supplementary Table S4.** mcBILAG improvement from week 4 through week 52 in patients with positive anti-dsDNA levels at baseline treated with belimumab versus placebo.

|  | BEL; n (%)  N=737 | PBO; n (%)  N=505 | OR | 95% CI | *p* value |
| --- | --- | --- | --- | --- | --- |
| **mcBILAG** **improvement** | | | | | |
| mcBILAG at week 4 | 275 (37.3) | 175 (34.7) | 1.16 | 0.91–1.47 | 0.235 |
| mcBILAG at week 8 | 358 (48.6) | 214 (42.4) | 1.23 | 0.98–1.56 | 0.076 |
| mcBILAG at week 12 | 394 (53.5) | 223 (44.2) | 1.45 | 1.15–1.83 | **0.001** |
| mcBILAG at week 16 | 413 (56.0) | 243 (48.1) | 1.33 | 1.06–1.68 | **0.015** |
| mcBILAG at week 20 | 419 (56.9) | 250 (49.5) | 1.32 | 1.05–1.66 | **0.020** |
| mcBILAG at week 24 | 436 (59.2) | 262 (51.9) | 1.29 | 1.02–1.63 | **0.031** |
| mcBILAG at week 28 | 434 (58.9) | 262 (51.9) | 1.30 | 1.03–1.64 | **0.026** |
| mcBILAG at week 32 | 437 (59.3) | 269 (53.3) | 1.21 | 0.96–1.53 | 0.101 |
| mcBILAG at week 36 | 432 (58.6) | 259 (51.3) | 1.31 | 1.04–1.65 | **0.022** |
| mcBILAG at week 40 | 447 (60.7) | 266 (52.7) | 1.34 | 1.06–1.69 | **0.014** |
| mcBILAG at week 44 | 445 (60.4) | 268 (53.1) | 1.31 | 1.04–1.65 | **0.024** |
| mcBILAG at week 48 | 451 (61.2) | 267 (52.9) | 1.36 | 1.08–1.71 | **0.010** |
| mcBILAG at week 52 | 459 (62.3) | 263 (52.1) | 1.44 | 1.14–1.82 | **0.002** |

Results from logistic regression, adjusted for trial variance with BLISS-52 and BLISS-76 pooled as a reference, of mcBILAG improvement from week 4 through week 52 in patients with positive anti-dsDNA levels at baseline treated with belimumab versus placebo. Data are presented as number of events (percentage), odds ratio (OR), 95% confidence interval (CI), and p value. Statistically significant p values are in bold.

Anti-dsDNA: anti double-stranded DNA antibodies; BEL: belimumab; mcBILAG: mucocutaneous British Isles Lupus Assessment Group; CI: confidence interval; NA: not applicable; OR: odds ratio; PBO: placebo.

# **Supplementary Table S5.** mcBILAG improvement from week 4 through week 52 in patients with negative anti-dsDNA levels at baseline treated with belimumab versus placebo.

|  | BEL; n (%)  N=350 | PBO; n (%)  N=247 | OR | 95% CI | *p* value |
| --- | --- | --- | --- | --- | --- |
| **mcBILAG** **improvement** | | | | | |
| mcBILAG at week 4 | 115 (32.9) | 82 (33.2) | 0.92 | 0.64–1.31 | 0.640 |
| mcBILAG at week 8 | 146 (41.7) | 95 (38.5) | 1.09 | 0.77–1.53 | 0.639 |
| mcBILAG at week 12 | 163 (46.6) | 113 (45.7) | 0.95 | 0.68–1.33 | 0.766 |
| mcBILAG at week 16 | 178 (50.9) | 124 (50.2) | 0.98 | 0.70–1.37 | 0.904 |
| mcBILAG at week 20 | 184 (52.6) | 111 (44.9) | 1.29 | 0.92–1.81 | 0.144 |
| mcBILAG at week 24 | 189 (54.0) | 130 (52.6) | 1.01 | 0.72–1.42 | 0.936 |
| mcBILAG at week 28 | 193 (55.1) | 126 (51.0) | 1.06 | 0.76–1.49 | 0.729 |
| mcBILAG at week 32 | 193 (55.1) | 133 (53.8) | 0.96 | 0.68–1.34 | 0.790 |
| mcBILAG at week 36 | 197 (56.3) | 132 (53.4) | 1.04 | 0.76–1.46 | 0.808 |
| mcBILAG at week 40 | 195 (55.7) | 137 (55.5) | 0.90 | 0.64–1.26 | 0.529 |
| mcBILAG at week 44 | 205 (58.6) | 137 (55.5) | 1.13 | 0.80–1.58 | 0.490 |
| mcBILAG at week 48 | 201 (57.4) | 130 (52.6) | 1.19 | 0.85–1.67 | 0.308 |
| mcBILAG at week 52 | 203 (58.0) | 140 (56.7) | 1.03 | 0.73–1.44 | 0.872 |

Results from logistic regression, adjusted for trial variance with BLISS-52 and BLISS-76 pooled as a reference, of mcBILAG improvement from week 4 through week 52 in patients with negative anti-dsDNA levels at baseline treated with belimumab versus placebo. Data are presented as number of events (percentage), odds ratio (OR), 95% confidence interval (CI), and *p* value. Statistically significant *p* values are in bold.

Anti-dsDNA: anti double-stranded DNA antibodies; BEL: belimumab; mcBILAG: mucocutaneous British Isles Lupus Assessment Group; CI: confidence interval; NA: not applicable: OR: odds ratio; PBO: placebo.

# **Supplementary Table S6.** mcBILAG improvement from week 4 through week 52 in patients with positive anti-dsDNA levels and/or low complement levels at baseline treated with belimumab versus placebo.

|  | BEL; n (%)  N=823 | PBO; n (%)  N=574 | OR | 95% CI | *p* value |
| --- | --- | --- | --- | --- | --- |
| **mcBILAG** **improvement** | | | | | |
| mcBILAG at week 4 | 299 (36.3) | 194 (33.8) | 1.15 | 0.91–1.44 | 0.244 |
| mcBILAG at week 8 | 395 (48.0) | 240 (41.8) | 1.24 | 0.99–1.54 | 0.058 |
| mcBILAG at week 12 | 439 (53.3) | 254 (44.3) | 1.43 | 1.15–1.78 | **0.001** |
| mcBILAG at week 16 | 462 (56.1) | 282 (49.1) | 1.30 | 1.04–1.61 | **0.019** |
| mcBILAG at week 20 | 470 (57.1) | 281 (49.0) | 1.36 | 1.09–1.69 | **0.006** |
| mcBILAG at week 24 | 485 (58.9) | 298 (51.9) | 1.29 | 1.03–1.60 | **0.023** |
| mcBILAG at week 28 | 484 (58.8) | 296 (51.6) | 1.31 | 1.05–1.63 | **0.016** |
| mcBILAG at week 32 | 492 (59.8) | 302 (52.6) | 1.27 | 1.02–1.58 | **0.030** |
| mcBILAG at week 36 | 487 (59.2) | 294 (51.2) | 1.35 | 1.09–1.68 | **0.007** |
| mcBILAG at week 40 | 502 (61.0) | 306 (53.3) | 1.33 | 1.06–1.65 | **0.012** |
| mcBILAG at week 44 | 497 (60.4) | 307 (53.5) | 1.30 | 1.04–1.62 | **0.019** |
| mcBILAG at week 48 | 505 (61.4) | 304 (53.0) | 1.37 | 1.10–1.71 | **0.004** |
| mcBILAG at week 52 | 510 (62.0) | 304 (53.0) | 1.39 | 1.11–1.73 | **0.003** |

Results from logistic regression, adjusted for trial variance with BLISS-52 and BLISS-76 pooled as a reference, of mcBILAG improvement from week 4 through week 52 in patients with positive anti-dsDNA levels and/or low complement levels at baseline treated with belimumab versus placebo. Data are presented as number of events (percentage), odds ratio (OR), 95% confidence interval (CI), and *p* value. Statistically significant *p* values are in bold.

Anti-dsDNA: anti double-stranded DNA antibodies; BEL: belimumab; mcBILAG: mucocutaneous British Isles Lupus Assessment Group; CI: confidence interval; NA: not applicable; OR: odds ratio; PBO: placebo.

# **Supplementary Table S7.** mcBILAG improvement from week 4 through week 52 in patients negative anti-dsDNA levels and normal/high complement levels at baseline treated with belimumab versus placebo.

|  | BEL; n (%)  N=264 | PBO; n (%)  N=178 | OR | 95% CI | *p* value |
| --- | --- | --- | --- | --- | --- |
| **mcBILAG** **improvement** | | | | | |
| mcBILAG at week 4 | 91 (34.5) | 63 (35.4) | 0.89 | 0.59–1.35 | 0.586 |
| mcBILAG at week 8 | 109 (41.3) | 69 (38.8) | 1.04 | 0.69–1.56 | 0.865 |
| mcBILAG at week 12 | 118 (44.7) | 82 (46.1) | 0.85 | 0.57–1.26 | 0.416 |
| mcBILAG at week 16 | 129 (48.9) | 85 (47.8) | 0.95 | 0.64–1.41 | 0.795 |
| mcBILAG at week 20 | 133 (50.4) | 80 (44.9) | 1.16 | 0.78–1.73 | 0.453 |
| mcBILAG at week 24 | 140 (53.0) | 94 (52.8) | 0.94 | 0.64–1.40 | 0.770 |
| mcBILAG at week 28 | 143 (54.2) | 92 (51.7) | 0.98 | 0.66–1.46 | 0.916 |
| mcBILAG at week 32 | 138 (52.3) | 100 (56.2) | 0.74 | 0.50–1.11 | 0.145 |
| mcBILAG at week 36 | 142 (53.8) | 97 (54.5) | 0.86 | 0.58–1.28 | 0.463 |
| mcBILAG at week 40 | 140 (53.0) | 97 (54.5) | 0.79 | 0.53–1.18 | 0.254 |
| mcBILAG at week 44 | 153 (58.0) | 98 (55.1) | 1.08 | 0.73–1.60 | 0.701 |
| mcBILAG at week 48 | 147 (55.7) | 93 (52.2) | 1.08 | 0.73–1.60 | 0.708 |
| mcBILAG at week 52 | 152 (57.6) | 99 (55.6) | 1.03 | 0.69–1.53 | 0.884 |

Results from logistic regression, adjusted for trial variance with BLISS-52 and BLISS-76 pooled as a reference, of mcBILAG improvement from week 4 through week 52 in patients with negative anti-dsDNA levels and normal/high complement levels at baseline treated with belimumab versus placebo. Data are presented as number of events (percentage), odds ratio (OR), 95% confidence interval (CI), and *p* value. Statistically significant *p* values are in bold.

Anti-dsDNA: anti double-stranded DNA antibodies; BEL: belimumab; mcBILAG: mucocutaneous British Isles Lupus Assessment Group; CI: confidence interval; NA: not applicable; OR: odds ratio; PBO: placebo.

# **Supplementary Table S8.** mcBILAG improvement from week 4 through week 52 in patients receiving prednisone equivalent dose > 7.5 mg/day at baseline treated with belimumab versus placebo.

|  | BEL; n (%)  N=684 | PBO; n (%)  N=472 | OR | 95% CI | *p* value |
| --- | --- | --- | --- | --- | --- |
| **mcBILAG** **improvement** | | | | | |
| mcBILAG at week 4 | 251 (36.7) | 163 (34.5) | 1.12 | 0.87–1.43 | 0.389 |
| mcBILAG at week 8 | 315 (46.1) | 203 (43.0) | 1.08 | 0.85–1.38 | 0.522 |
| mcBILAG at week 12 | 348 (50.9) | 212 (44.9) | 1.26 | 0.99–1.60 | 0.059 |
| mcBILAG at week 16 | 368 (53.8) | 229 (48.5) | 1.20 | 0.95–1.53 | 0.131 |
| mcBILAG at week 20 | 380 (55.6) | 225 (47.7) | 1.34 | 1.05–1.70 | **0.017** |
| mcBILAG at week 24 | 401 (58.6) | 247 (52.3) | 1.25 | 0.98–1.59 | 0.070 |
| mcBILAG at week 28 | 397 (58.0) | 245 (51.9) | 1.21 | 0.95–1.54 | 0.117 |
| mcBILAG at week 32 | 398 (58.2) | 257 (54.4) | 1.10 | 0.87–1.40 | 0.434 |
| mcBILAG at week 36 | 404 (59.1) | 252 (53.4) | 1.21 | 0.95–1.54 | 0.114 |
| mcBILAG at week 40 | 408 (59.6) | 257 (54.4) | 1.18 | 0.93–1.51 | 0.168 |
| mcBILAG at week 44 | 412 (60.2) | 257 (54.4) | 1.24 | 0.97–1.57 | 0.084 |
| mcBILAG at week 48 | 418 (61.1) | 254 (53.8) | 1.31 | 1.03–1.67 | **0.027** |
| mcBILAG at week 52 | 416 (60.8) | 256 (54.2) | 1.24 | 0.98–1.58 | 0.077 |

Results from logistic regression, adjusted for trial variance with BLISS-52 and BLISS-76 pooled as a reference, of mcBILAG improvement from week 4 through week 52 in patients receiving prednisone equivalent dose > 7.5 mg/day at baseline treated with belimumab versus placebo. Data are presented as number of events (percentage), odds ratio (OR), 95% confidence interval (CI), and *p* value. Statistically significant *p* values are in bold.

BEL: belimumab; mcBILAG: mucocutaneous British Isles Lupus Assessment Group; CI: confidence interval; NA: not applicable; OR: odds ratio; PBO: placebo.

# **Supplementary Table S9.** mcBILAG improvement from week 4 through week 52 in patients receiving prednisone equivalent dose ≤ 7.5 mg/day at baseline treated with belimumab versus placebo.

|  | BEL; n (%)  N=403 | PBO; n (%)  N=280 | OR | 95% CI | *p* value |
| --- | --- | --- | --- | --- | --- |
| **mcBILAG** **improvement** | | | | | |
| mcBILAG at week 4 | 139 (34.5) | 94 (33.6) | 1.02 | 0.73–1.42 | 0.930 |
| mcBILAG at week 8 | 189 (46.9) | 106 (37.9) | 1.41 | 1.02–1.94 | **0.036** |
| mcBILAG at week 12 | 209 (51.9) | 124 (44.3) | 1.30 | 0.95–1.78 | 0.105 |
| mcBILAG at week 16 | 223 (55.3) | 138 (49.3) | 1.22 | 0.89–1.68 | 0.209 |
| mcBILAG at week 20 | 223 (55.3) | 136 (48.6) | 1.27 | 0.92–1.73 | 0.143 |
| mcBILAG at week 24 | 224 (55.6) | 145 (51.8) | 1.13 | 0.82–1.54 | 0.463 |
| mcBILAG at week 28 | 230 (57.1) | 143 (51.1) | 1.28 | 0.93–1.76 | 0.123 |
| mcBILAG at week 32 | 232 (57.6) | 145 (51.8) | 1.19 | 0.87–1.63 | 0.276 |
| mcBILAG at week 36 | 225 (55.8) | 139 (49.6) | 1.26 | 0.92–1.73 | 0.147 |
| mcBILAG at week 40 | 234 (58.1) | 146 (52.1) | 1.19 | 0.87–1.63 | 0.287 |
| mcBILAG at week 44 | 238 (59.1) | 148 (52.9) | 1.29 | 0.94–1.78 | 0.110 |
| mcBILAG at week 48 | 234 (58.1) | 143 (51.1) | 1.30 | 0.95–1.79 | 0.099 |
| mcBILAG at week 52 | 246 (61.0) | 147 (52.5) | 1.42 | 1.03–1.94 | **0.032** |

Results from logistic regression, adjusted for trial variance with BLISS-52 and BLISS-76 pooled as a reference, of mcBILAG improvement from week 4 through week 52 in patients receiving prednisone equivalent dose ≤ 7.5 mg/day at baseline treated with belimumab versus placebo. Data are presented as number of events (percentage), odds ratio (OR), 95% confidence interval (CI), and *p* value. Statistically significant *p* values are in bold.

BEL: belimumab; mcBILAG: mucocutaneous British Isles Lupus Assessment Group; CI: confidence interval; NA: not applicable; OR: odds ratio; PBO: placebo.

# **Supplementary Table S10.** mcSLEDAI-2K improvement from week 4 through week 52 in patients with SLE treated with belimumab versus placebo in the pooled belimumab RCT population.

|  | BEL; n (%)  N=1585 | PBO; n (%)  N=1039 | OR | 95% CI | *p* value |
| --- | --- | --- | --- | --- | --- |
| **mcSLEDAI-2K improvement** | | | | | |
| mcSLEDAI-2K at week 4 | 383 (24.2) | 249 (24.0) | 1.04 | 0.86–1.25 | 0.669 |
| mcSLEDAI-2K at week 8 | 612 (38.6) | 369 (35.5) | 1.17 | 0.99–1.38 | 0.061 |
| mcSLEDAI-2K at week 12 | 757 (47.8) | 459 (44.2) | 1.15 | 0.98–1.34 | 0.096 |
| mcSLEDAI-2K at week 16 | 848 (53.5) | 502 (48.3) | 1.20 | 1.02–1.41 | **0.025** |
| mcSLEDAI-2K at week 20 | 911 (57.5) | 557 (53.6) | 1.15 | 0.98–1.34 | 0.096 |
| mcSLEDAI-2K at week 24 | 978 (61.7) | 582 (56.0) | 1.24 | 1.05–1.45 | **0.010** |
| mcSLEDAI-2K at week 28 | 998 (63.0) | 606 (58.3) | 1.18 | 1.00–1.39 | **0.045** |
| mcSLEDAI-2K at week 32 | 1016 (64.1) | 609 (58.6) | 1.23 | 1.04–1.45 | **0.013** |
| mcSLEDAI-2K at week 36 | 1043 (65.8) | 607 (58.4) | 1.33 | 1.13–1.57 | **0.001** |
| mcSLEDAI-2K at week 40 | 1048 (66.1) | 614 (59.1) | 1.30 | 1.11–1.54 | **0.001** |
| mcSLEDAI-2K at week 44 | 1075 (67.8) | 617 (59.4) | 1.39 | 1.18–1.64 | **< 0.001** |
| mcSLEDAI-2K at week 48 | 1089 (68.7) | 626 (60.3) | 1.40 | 1.18–1.65 | **< 0.001** |
| mcSLEDAI-2K at week 52 | 1102 (69.5) | 638 (61.4) | 1.37 | 1.16–1.62 | **< 0.001** |

Results from logistic regression, adjusted for trial variance with BLISS-52 and BLISS-76 pooled as a reference, of mcSLEDAI-2K improvement from week 4 through week 52 in patients with SLE treated with belimumab versus placebo in the pooled belimumab RCT population. Data are presented as number of events (percentage), odds ratio (OR), 95% confidence interval (CI), and *p* value. Statistically significant *p* values are in bold.

BEL: belimumab; mcSLEDAI-2K: mucocutaneous Systemic Lupus Erythematosus Disease Activity Index 2000; CI: confidence interval; NA: not applicable; OR: odds ratio; PBO: placebo; RCT: randomized controlled trial.

# **Supplementary Table S11.** mcSLEDAI-2K improvement from week 4 through week 52 in patients with SLEDAI-2K ≥ 10 at baseline treated with belimumab versus placebo.

|  | BEL; n (%)  N=1018 | PBO; n (%)  N=654 | OR | 95% CI | *p* value |
| --- | --- | --- | --- | --- | --- |
| **mcSLEDAI-2K improvement** | | | | | |
| mcSLEDAI-2K at week 4 | 263 (25.8) | 166 (25.4) | 1.06 | 0.84–1.33 | 0.629 |
| mcSLEDAI-2K at week 8 | 419 (41.2) | 235 (35.9) | 1.28 | 1.04–1.57 | **0.019** |
| mcSLEDAI-2K at week 12 | 522 (51.3) | 290 (44.3) | 1.30 | 1.07–1.59 | **0.010** |
| mcSLEDAI-2K at week 16 | 574 (56.4) | 318 (48.6) | 1.31 | 1.07–1.60 | **0.008** |
| mcSLEDAI-2K at week 20 | 610 (59.9) | 353 (54.0) | 1.23 | 1.01–1.51 | **0.041** |
| mcSLEDAI-2K at week 24 | 661 (64.9) | 370 (56.6) | 1.38 | 1.12–1.69 | **0.002** |
| mcSLEDAI-2K at week 28 | 663 (65.1) | 383 (58.6) | 1.27 | 1.03–2.59 | **0.023** |
| mcSLEDAI-2K at week 32 | 673 (66.1) | 384 (58.7) | 1.33 | 1.09–1.64 | **0.006** |
| mcSLEDAI-2K at week 36 | 692 (68.0) | 382 (58.4) | 1.47 | 1.19–1.80 | **< 0.001** |
| mcSLEDAI-2K at week 40 | 687 (67.5) | 389 (59.5) | 1.37 | 1.11–1.68 | **0.003** |
| mcSLEDAI-2K at week 44 | 707 (69.4) | 384 (58.7) | 1.54 | 1.25–1.90 | **< 0.001** |
| mcSLEDAI-2K at week 48 | 716 (70.3) | 394 (60.2) | 1.51 | 1.23–1.87 | **< 0.001** |
| mcSLEDAI-2K at week 52 | 723 (71.0) | 400 (61.2) | 1.49 | 1.20–1.84 | **< 0.001** |

Results from logistic regression, adjusted for trial variance with BLISS-52 and BLISS-76 pooled as a reference, of mcSLEDAI-2K improvement from week 4 through week 52 in patients with SLEDAI-2K score ≥ 10 at baseline treated with belimumab versus placebo. Data are presented as number of events (percentage), odds ratio (OR), 95% confidence interval (CI), and *p* value. Statistically significant *p* values are in bold.

BEL: belimumab; mcSLEDAI-2K: mucocutaneous Systemic Lupus Erythematosus Disease Activity Index 2000; CI: confidence interval; NA: not applicable; OR: odds ratio; PBO: placebo; SLEDAI-2K: Systemic Lupus Erythematosus Disease Activity Index 2000.

# **Supplementary Table S12.** mcSLEDAI-2K improvement from week 4 through week 52 in patients with SLEDAI-2K < 10 at baseline treated with belimumab versus placebo.

|  | BEL; n (%)  N=567 | | PBO; n (%)  N=385 | OR | 95% CI | *p* value |
| --- | --- | --- | --- | --- | --- | --- |
| **mcSLEDAI-2K improvement** | | | | | | |
| mcSLEDAI-2K at week 4 | | 120 (21.2) | 83 (21.6) | 1.02 | 0.74–1.41 | 0.905 |
| mcSLEDAI-2K at week 8 | | 193 (34.0) | 134 (34.8) | 1.00 | 0.76–1.32 | 0.983 |
| mcSLEDAI-2K at week 12 | | 235 (41.4) | 169 (43.9) | 0.92 | 0.70–1.20 | 0.525 |
| mcSLEDAI-2K at week 16 | | 274 (48.3) | 184 (47.8) | 1.02 | 0.79–1.33 | 0.859 |
| mcSLEDAI-2K at week 20 | | 301 (53.1) | 204 (53.0) | 1.00 | 0.77–1.30 | 0.989 |
| mcSLEDAI-2K at week 24 | | 317 (55.9) | 212 (55.1) | 1.03 | 0.79–1. 34 | 0.818 |
| mcSLEDAI-2K at week 28 | | 335 (59.1) | 223 (57.9) | 1.05 | 0.80–1. 37 | 0.744 |
| mcSLEDAI-2K at week 32 | | 343 (60.5) | 225 (58.4) | 1.06 | 0.81–1. 39 | 0.650 |
| mcSLEDAI-2K at week 36 | | 343 (60.5) | 225 (58.4) | 1.12 | 0.85–1.46 | 0.424 |
| mcSLEDAI-2K at week 40 | | 361 (63.7) | 225 (58.4) | 1.19 | 0.91–1.56 | 0.209 |
| mcSLEDAI-2K at week 44 | | 368 (64.9) | 233 (60.5) | 1.16 | 0.88–1.53 | 0.282 |
| mcSLEDAI-2K at week 48 | | 373 (65.8) | 232 (60.3) | 1.22 | 0.93.–1.60 | 0.160 |
| mcSLEDAI-2K at week 52 | | 379 (66.8) | 238 (61.8) | 1.20 | 0.91–1.58 | 0.198 |

Results from logistic regression, adjusted for trial variance with BLISS-52 and BLISS-76 pooled as a reference, of mcSLEDAI-2K improvement from week 4 through week 52 in patients with SLEDAI-2K score < 10 at baseline treated with belimumab versus placebo. Data are presented as number of events (percentage), odds ratio (OR), 95% confidence interval (CI), and *p* value. Statistically significant *p* values are in bold.

BEL: belimumab; mcSLEDAI-2K: mucocutaneous Systemic Lupus Erythematosus Disease Activity Index 2000; CI: confidence interval; NA: not applicable; OR: odds ratio; PBO: placebo; SLEDAI-2K: Systemic Lupus Erythematosus Disease Activity Index 2000.

# **Supplementary Table S13.** mcSLEDAI-2K improvement from week 4 through week 52 in patients with positive anti-dsDNA levels at baseline treated with belimumab versus placebo.

|  | BEL; n (%)  N=1106 | | PBO; n (%)  N=713 | OR | 95% CI | *p* value |
| --- | --- | --- | --- | --- | --- | --- |
| **mcSLEDAI-2K improvement** | | | | | | |
| mcSLEDAI-2K at week 4 | | 261 (23.6) | 166 (23.3) | 1.05 | 0.84–1.31 | 0.691 |
| mcSLEDAI-2K at week 8 | | 427 (38.6) | 241 (33.8) | 1.27 | 1.04–1.55 | **0.020** |
| mcSLEDAI-2K at week 12 | | 517 (46.7) | 302 (42.4) | 1.19 | 0.98–1.44 | 0.080 |
| mcSLEDAI-2K at week 16 | | 580 (52.4) | 325 (45.6) | 1.28 | 1.06–1.55 | **0.011** |
| mcSLEDAI-2K at week 20 | | 622 (56.2) | 369 (51.8) | 1.18 | 0.97–1.42 | 0.097 |
| mcSLEDAI-2K at week 24 | | 679 (61.4) | 384 (53.9) | 1.33 | 1.10–1.62 | **0.004** |
| mcSLEDAI-2K at week 28 | | 691 (62.5) | 397 (55.7) | 1.29 | 1.06–1.57 | **0.010** |
| mcSLEDAI-2K at week 32 | | 701 (63.4) | 403 (56.5) | 1.31 | 1.08–1.59 | **0.006** |
| mcSLEDAI-2K at week 36 | | 717 (64.8) | 397 (55.7) | 1.44 | 1.19–1.75 | **<0.001** |
| mcSLEDAI-2K at week 40 | | 724 (65.5) | 404 (56.7) | 1.41 | 1.16–1.72 | **0.001** |
| mcSLEDAI-2K at week 44 | | 739 (66.8) | 403 (56.5) | 1.51 | 1.24–1.84 | **<0.001** |
| mcSLEDAI-2K at week 48 | | 748 (67.6) | 412 (57.8) | 1.49 | 1.22–1.81 | **<0.001** |
| mcSLEDAI-2K at week 52 | | 757 (68.4) | 419 (58.8) | 1.47 | 1.20–1.79 | **<0.001** |

Results from logistic regression, adjusted for trial variance with BLISS-52 and BLISS-76 pooled as a reference, of mcSLEDAI-2K improvement from week 4 through week 52 in patients with positive anti-dsDNA levels at baseline treated with belimumab versus placebo. Data are presented as number of events (percentage), odds ratio (OR), 95% confidence interval (CI), and *p* value. Statistically significant *p* values are in bold.

Anti-dsDNA: anti double-stranded DNA antibodies; BEL: belimumab; mcSLEDAI-2K: mucocutaneous Systemic Lupus Erythematosus Disease Activity Index 2000; CI: confidence interval; NA: not applicable; OR: odds ratio; PBO: placebo.

# **Supplementary Table S14.** mcSLEDAI-2K improvement from week 4 through week 52 in patients with negative anti-dsDNA levels at baseline treated with belimumab versus placebo.

|  | BEL; n (%)  N=479 | | PBO; n (%)  N=326 | OR | 95% CI | *p* value |
| --- | --- | --- | --- | --- | --- | --- |
| **mcSLEDAI-2K improvement** | | | | | | |
| mcSLEDAI-2K at week 4 | | 122 (25.5) | 83 (25.5) | 1.02 | 0.73–1.42 | 0.911 |
| mcSLEDAI-2K at week 8 | | 185 (38.6) | 128 (39.3) | 0.97 | 0.72–1.31 | 0.850 |
| mcSLEDAI-2K at week 12 | | 240 (50.1) | 157 (48.2) | 1.05 | 0.78–1.39 | 0.759 |
| mcSLEDAI-2K at week 16 | | 268 (55.9) | 177 (54.3) | 1.02 | 0.76–1.36 | 0.896 |
| mcSLEDAI-2K at week 20 | | 289 (60.3) | 188 (57.7) | 1.06 | 0.79–1.42 | 0.696 |
| mcSLEDAI-2K at week 24 | | 299 (62.4) | 198 (60.7) | 1.03 | 0.76–1.38 | 0.863 |
| mcSLEDAI-2K at week 28 | | 307 (64.1) | 209 (64.1) | 0.94 | 0.70–1.27 | 0.695 |
| mcSLEDAI-2K at week 32 | | 315 (65.8) | 206 (63.2) | 1.03 | 0.77–1.40 | 0.825 |
| mcSLEDAI-2K at week 36 | | 326 (68.1) | 210 (64.4) | 1.09 | 0.80–1.47 | 0.596 |
| mcSLEDAI-2K at week 40 | | 324 (67.6) | 210 (64.4) | 1.06 | 0.78–1.44 | 0.696 |
| mcSLEDAI-2K at week 44 | | 336 (70.1) | 214 (65.6) | 1.14 | 0.84–1.55 | 0.411 |
| mcSLEDAI-2K at week 48 | | 341 (71.2) | 214 (65.6) | 1.20 | 0.88–1.63 | 0.255 |
| mcSLEDAI-2K at week 52 | | 345 (72.0) | 219 (67.2) | 1.16 | 0.85–1.58 | 0.360 |

Results from logistic regression, adjusted for trial variance with BLISS-52 and BLISS-76 pooled as a reference, of mcSLEDAI-2K improvement from week 4 through week 52 in patients with negative anti-dsDNA levels at baseline treated with belimumab versus placebo. Data are presented as number of events (percentage), odds ratio (OR), 95% confidence interval (CI), and *p* value. Statistically significant *p* values are in bold.

Anti-dsDNA: anti double-stranded DNA antibodies; BEL: belimumab; mcSLEDAI-2K: mucocutaneous Systemic Lupus Erythematosus Disease Activity Index 2000; CI: confidence interval; NA: not applicable: OR: odds ratio; PBO: placebo.

# **Supplementary Table S15.** mcSLEDAI-2K improvement from week 4 through week 52 in patients with positive anti-dsDNA levels and/or low complement levels at baseline treated with belimumab versus placebo.

|  | BEL; n (%)  N=1230 | | PBO; n (%)  N=806 | OR | 95% CI | *p* value |
| --- | --- | --- | --- | --- | --- | --- |
| **mcSLEDAI-2K improvement** | | | | | | |
| mcSLEDAI-2K at week 4 | | 292 (23.7) | 184 (22.8) | 1.07 | 0.87–1.33 | 0.507 |
| mcSLEDAI-2K at week 8 | | 473 (38.5) | 272 (33.7) | 1.25 | 1.04–1.51 | **0.020** |
| mcSLEDAI-2K at week 12 | | 580 (47.2) | 338 (41.9) | 1.23 | 1.02–1.47 | **0.027** |
| mcSLEDAI-2K at week 16 | | 653 (53.1) | 370 (45.9) | 1.31 | 1.09–1.56 | **0.004** |
| mcSLEDAI-2K at week 20 | | 696 (56.6) | 420 (52.1) | 1.18 | 0.98–1.41 | 0.074 |
| mcSLEDAI-2K at week 24 | | 758 (61.6) | 437 (54.2) | 1.33 | 1.11–1.59 | **0.002** |
| mcSLEDAI-2K at week 28 | | 769 (62.5) | 450 (55.8) | 1.29 | 1.07–1.55 | **0.007** |
| mcSLEDAI-2K at week 32 | | 782 (63.6) | 454 (56.3) | 1.34 | 1.11–1.61 | **0.002** |
| mcSLEDAI-2K at week 36 | | 803 (65.3) | 448 (55.6) | 1.47 | 1.23–1.77 | **<0.001** |
| mcSLEDAI-2K at week 40 | | 809 (65.8) | 456 (56.6) | 1.44 | 1.19–1.73 | **<0.001** |
| mcSLEDAI-2K at week 44 | | 826 (67.2) | 456 (56.6) | 1.53 | 1.27–1.84 | **<0.001** |
| mcSLEDAI-2K at week 48 | | 836 (68.0) | 466 (57.8) | 1.51 | 1.25–1.82 | **<0.001** |
| mcSLEDAI-2K at week 52 | | 847 (68.9) | 474 (58.8) | 1.49 | 1.24–1.80 | **<0.001** |

Results from logistic regression, adjusted for trial variance with BLISS-52 and BLISS-76 pooled as a reference, of mcSLEDAI-2K improvement from week 4 through week 52 in patients with positive anti-dsDNA levels and/or low complement levels at baseline treated with belimumab versus placebo. Data are presented as number of events (percentage), odds ratio (OR), 95% confidence interval (CI), and *p* value. Statistically significant *p* values are in bold.

Anti-dsDNA: anti double-stranded DNA antibodies; BEL: belimumab; mcSLEDAI-2K: mucocutaneous Systemic Lupus Erythematosus Disease Activity Index 2000; CI: confidence interval; NA: not applicable; OR: odds ratio; PBO: placebo.

# **Supplementary Table S16.** mcSLEDAI-2K improvement from week 4 through week 52 in patients with negative anti-dsDNA levels and normal/high complement levels at baseline treated with belimumab versus placebo.

|  | BEL; n (%)  N=355 | | PBO; n (%)  N=233 | OR | 95% CI | *p* value |
| --- | --- | --- | --- | --- | --- | --- |
| **mcSLEDAI-2K improvement** | | | | | | |
| mcSLEDAI-2K at week 4 | | 91 (25.6) | 65 (27.9) | 0.95 | 0.65–1.39 | 0.777 |
| mcSLEDAI-2K at week 8 | | 139 (39.2) | 97 (41.6) | 0.94 | 0.67–1.33 | 0.747 |
| mcSLEDAI-2K at week 12 | | 177 (49.9) | 121 (51.9) | 0.90 | 0.64–1.26 | 0.541 |
| mcSLEDAI-2K at week 16 | | 195 (54.9) | 132 (56.7) | 0.88 | 0.62–1.23 | 0.452 |
| mcSLEDAI-2K at week 20 | | 215 (60.6) | 137 (58.8) | 1.02 | 0.72–1.44 | 0.923 |
| mcSLEDAI-2K at week 24 | | 220 (62.0) | 145 (62.2) | 0.94 | 0.67–1.34 | 0.750 |
| mcSLEDAI-2K at week 28 | | 229 (64.5) | 156 (67.0) | 0.85 | 0.59–1.22 | 0.376 |
| mcSLEDAI-2K at week 32 | | 234 (65.9) | 155 (66.5) | 0.88 | 0.61–1.26 | 0.482 |
| mcSLEDAI-2K at week 36 | | 240 (67.6) | 159 (68.2) | 0.89 | 0.62–1.28 | 0.537 |
| mcSLEDAI-2K at week 40 | | 239 (67.3) | 158 (67.8) | 0.90 | 0.62–1.29 | 0.554 |
| mcSLEDAI-2K at week 44 | | 249 (70.1) | 161 (69.1) | 0.98 | 0.67–1.41 | 0.898 |
| mcSLEDAI-2K at week 48 | | 253 (71.3) | 160 (68.7) | 1.04 | 0.72–1.50 | 0.841 |
| mcSLEDAI-2K at week 52 | | 255 (71.8) | 164 (70.4) | 0.98 | 0.67–1.42 | 0.908 |

Results from logistic regression, adjusted for trial variance with BLISS-52 and BLISS-76 pooled as a reference, of mcSLEDAI-2K improvement from week 4 through week 52 in patients with negative anti-dsDNA levels and normal/high complement levels at baseline treated with belimumab versus placebo. Data are presented as number of events (percentage), odds ratio (OR), 95% confidence interval (CI), and *p* value. Statistically significant *p* values are in bold.

Anti-dsDNA: anti double-stranded DNA antibodies; BEL: belimumab; mcSLEDAI-2K: mucocutaneous Systemic Lupus Erythematosus Disease Activity Index 2000; CI: confidence interval; NA: not applicable; OR: odds ratio; PBO: placebo.

# **Supplementary Table S17.** mcSLEDAI-2K improvement from week 4 through week 52 in patients receiving prednisone equivalent dose > 7.5 mg/day at baseline treated with belimumab versus placebo.

|  | BEL; n (%)  N=1006 | | PBO; n (%)  N=659 | OR | 95% CI | *p* value |
| --- | --- | --- | --- | --- | --- | --- |
| **mcSLEDAI-2K improvement** | | | | | | |
| mcSLEDAI-2K at week 4 | | 234 (23.3) | 171 (25.9) | 0.89 | 0.71–1.13 | 0.340 |
| mcSLEDAI-2K at week 8 | | 382 (38.0) | 245 (37.2) | 1.06 | 0.87–1.31 | 0.555 |
| mcSLEDAI-2K at week 12 | | 489 (48.6) | 306 (46.4) | 1.09 | 0.89–1.33 | 0.398 |
| mcSLEDAI-2K at week 16 | | 547 (54.4) | 333 (50.5) | 1.14 | 0.93–1.39 | 0.197 |
| mcSLEDAI-2K at week 20 | | 589 (58.5) | 365 (55.4) | 1.11 | 0.91–1.36 | 0.303 |
| mcSLEDAI-2K at week 24 | | 634 (63.0) | 391 (59.3) | 1.14 | 0.93–1.40 | 0.215 |
| mcSLEDAI-2K at week 28 | | 648 (64.4) | 401 (60.8) | 1.13 | 0.92–1.39 | 0.229 |
| mcSLEDAI-2K at week 32 | | 661 (65.7) | 402 (61.0) | 1.20 | 0.98–1.48 | 0.078 |
| mcSLEDAI-2K at week 36 | | 676 (67.2) | 402 (61.0) | 1.29 | 1.04–1.58 | **0.018** |
| mcSLEDAI-2K at week 40 | | 681 (67.7) | 405 (61.5) | 1.28 | 1.04–1.58 | **0.018** |
| mcSLEDAI-2K at week 44 | | 698 (69.4) | 413 (62.7) | 1.31 | 1.06–1.61 | **0.012** |
| mcSLEDAI-2K at week 48 | | 706 (70.2) | 418 (63.4) | 1.33 | 1.07–1.64 | **0.009** |
| mcSLEDAI-2K at week 52 | | 715 (71.1) | 419 (63.6) | 1.36 | 1.10–1.68 | **0.004** |

Results from logistic regression, adjusted for trial variance with BLISS-52 and BLISS-76 pooled as a reference, of mcSLEDAI-2K improvement from week 4 through week 52 in patients receiving prednisone equivalent dose > 7.5 mg/day at baseline treated with belimumab versus placebo. Data are presented as number of events (percentage), odds ratio (OR), 95% confidence interval (CI), and *p* value. Statistically significant *p* values are in bold.

BEL: belimumab; mcSLEDAI-2K: mucocutaneous Systemic Lupus Erythematosus Disease Activity Index 2000; CI: confidence interval; NA: not applicable; OR: odds ratio; PBO: placebo.

# **Supplementary Table S18.** mcSLEDAI-2K improvement from week 4 through week 52 in patients receiving prednisone equivalent dose ≤ 7.5 mg/day at baseline treated with belimumab versus placebo.

|  | BEL; n (%)  N=579 | | PBO; n (%)  N=380 | OR | 95% CI | *p* value |
| --- | --- | --- | --- | --- | --- | --- |
| **mcSLEDAI-2K improvement** | | | | | | |
| mcSLEDAI-2K at week 4 | | 149 (25.7) | 78 (20.5) | 1.38 | 1.01–1.90 | **0.045** |
| mcSLEDAI-2K at week 8 | | 230 (39.7) | 124 (32.6) | 1.40 | 1.06–1.85 | **0.017** |
| mcSLEDAI-2K at week 12 | | 268 (46.3) | 153 (40.3) | 1.27 | 0.97–1.66 | 0.084 |
| mcSLEDAI-2K at week 16 | | 301 (52.0) | 169 (44.5) | 1.33 | 1.02–1.73 | **0.037** |
| mcSLEDAI-2K at week 20 | | 322 (55.6) | 192 (50.5) | 1.23 | 0.94–1.60 | 0.131 |
| mcSLEDAI-2K at week 24 | | 344 (59.4) | 191 (50.3) | 1.45 | 1.11–1.89 | **0.006** |
| mcSLEDAI-2K at week 28 | | 350 (60.4) | 205 (53.9) | 1.29 | 0.98–1.68 | 0.065 |
| mcSLEDAI-2K at week 32 | | 355 (61.3) | 207 (54.5) | 1.29 | 0.99–1.69 | 0.063 |
| mcSLEDAI-2K at week 36 | | 367 (63.4) | 205 (53.9) | 1.43 | 1.09–1.87 | **0.009** |
| mcSLEDAI-2K at week 40 | | 367 (63.4) | 209 (55.0) | 1.36 | 1.04–1.78 | **0.025** |
| mcSLEDAI-2K at week 44 | | 377 (65.1) | 204 (53.7) | 1.58 | 1.20–2.07 | **0.001** |
| mcSLEDAI-2K at week 48 | | 383 (66.1) | 208 (54.7) | 1.55 | 1.18–2.04 | **0.002** |
| mcSLEDAI-2K at week 52 | | 387 (66.8) | 219 (57.6) | 1.42 | 1.08–1.86 | **0.013** |

Results from logistic regression, adjusted for trial variance with BLISS-52 and BLISS-76 pooled as a reference, of mcSLEDAI-2K improvement from week 4 through week 52 in patients receiving prednisone equivalent dose ≤ 7.5 mg/day at baseline treated with belimumab versus placebo. Data are presented as number of events (percentage), odds ratio (OR), 95% confidence interval (CI), and *p* value. Statistically significant *p* values are in bold.

BEL: belimumab; mcSLEDAI-2K: mucocutaneous Systemic Lupus Erythematosus Disease Activity Index 2000; CI: confidence interval; NA: not applicable; OR: odds ratio; PBO: placebo.
